# Confidence-weighted Testing as an Impactful Education Intervention within a Pediatric Sepsis Quality Improvement Initiative

**DOI:** 10.1097/pq9.0000000000000460

**Published:** 2021-08-26

**Authors:** Emma D. Nathaniel, Halden F. Scott, Beth Wathen, Sarah K. Schmidt, Elise Rolison, Carter Smith, Matthew J. Hays, Justin M. Lockwood

**Affiliations:** From the * Section of Hospital Medicine, Department of Pediatrics, University of Colorado School of Medicine, Aurora, Colo.; † Section of Emergency Medicine, Department of Pediatrics, University of Colorado School of Medicine, Aurora, Colo.; ‡ Pediatric Intensive Care Unit, Children’s Hospital Colorado, Aurora, Colo.; § Clinical Effectiveness, Children’s Hospital Colorado, Aurora, Colo.; ¶ Amplifire, Inc., Boulder, Colo.

## Abstract

**Introduction:**

Confidence-weighted testing assesses learners’ beliefs about their knowledge and skills. As part of a hospital-wide quality improvement initiative to enhance care for pediatric patients with suspected sepsis, we developed a novel intervention using confidence-weighted testing to identify institutional areas of misinformation and knowledge gaps while also providing real-time feedback to individual learners.

**Methods:**

We developed pediatric sepsis eLearning modules incorporating confidence-weighted testing. We distributed them to nurses, advanced practitioners, and physicians in emergency departments and acute care/non-intensive care unit inpatient settings in our hospital system. We analyzed completion and response data over 2 years following module distribution. Our outcomes included completion, *confidently held misinformation* (CHM; when a learner answers a question confidently but incorrectly), *struggle* (when a learner repeatedly answers a question incorrectly or with low confidence), and *mastery* (when a learner initially answers a question correctly and confidently).

**Results:**

Eighty-three percent of assigned learners completed the modules (1,463/1,754). Although nurses had significantly more misinformation and struggled more than physicians and advanced practitioners, learners of all roles achieved 100% mastery as part of module completion. The greatest CHM and struggle were found in serum lactate interpretation’s nuances and the hemodynamic shock states commonly seen in sepsis.

**Conclusions:**

Our novel application of confidence-weighted testing enhanced learning by correcting learners’ misinformation. It also identified systems issues and institutional knowledge gaps as targets for future improvement.

## Abstract 2

Supplemental Digital Content is available in the text.

## INTRODUCTION

Sepsis is a leading cause of mortality in children,^1^ and studies have attributed preventable deaths from sepsis to deficits in its recognition and management.^2^ Experts emphasize the importance of standardized approaches to sepsis care to improve patient outcomes.^3,4^ Quality improvement (QI) initiatives enhance sepsis care,^5,6^ and the use of electronic learning (eLearning) is a common component of these initiatives.^7^

Our institution engaged in a hospital-wide QI initiative to improve the care provided to patients with suspected sepsis in the emergency department (ED) and acute care (non-intensive care unit [ICU]) inpatient settings. We identified sepsis education as a key driver in our initiative. We developed a novel sepsis eLearning intervention that allowed for real-time feedback to the learner to correct misinformation and address knowledge gaps. The eLearning tool also captured learners’ confidence to identify and remediate *confidently held misinformation* (CHM). We focused on confidence because when someone is sure they are correct, they are more likely to act without consulting guidelines or peers.^8,9^ When this confidence is misplaced, providers’ actions can put patients at greater risk.

This project’s objectives were to (1) provide education on pediatric sepsis to frontline care-team members while introducing them to our QI interventions and (2) identify systems issues to highlight areas of opportunity for ongoing improvement. Although other studies have demonstrated the value of eLearning in pediatric sepsis,^7^ this report aims to describe our experience applying confidence-weighted testing to enhance learning.^10,11^ To the extent that our initiative was successful, the principles we used in developing and evaluating our intervention could be incorporated in future education components of other QI initiatives to optimize learning and identify specific areas of local misinformation that could inform subsequent improvement efforts.

## METHODS

The study was approved for conduct as nonhuman subject’s research by the study site’s Organizational Research Risk and Quality Improvement Review Panel.

### Context

The study occurred at a free-standing, quaternary care, academic children’s hospital with on-site ICUs (pediatric, cardiac, and neonatal). The hospital system also incorporates 3 smaller community hospitals without on-site ICUs. The admitting acute care/non-ICU services at our largest hospital include general pediatrics/hospital medicine, hematology/oncology, pulmonology, gastroenterology/liver, surgical subspecialties, and several other medical subspecialties (for the remainder of this report, we refer to these services/units as “acute care”). Bedside nurses generally work in specific units, with only a small subset floating between units. Pediatric residents rotate on all ED and nonsurgical acute care units for 1 month at a time. The institution’s sepsis initiative has focused efforts on the ED and acute care inpatient units.

### Intervention

Local pediatric sepsis experts developed the educational content by reviewing national pediatric sepsis guidelines,^3^ published sepsis educational goals,^12^ and institution-specific sepsis practices. We developed the modules in collaboration with a proprietary eLearning platform (Amplifire, Boulder, Colo.) and housed them within our institutional learning management system (LMS). Amplifire is an eLearning platform that uses an adaptive algorithm to detect and correct misinformation and knowledge gaps in learners within question-based educational modules. Our LMS (Cornerstone, Santa Monica, Calif.) facilitates online courses and registration for instructor-led training. For this module, the online training through Amplifire was assigned to individual learners and monitored for completion within our LMS.

We created 3 modules to best address the differences in practice between clinical units: (1) ED module; (2) acute care inpatient module; and (3) pediatric resident module (hybrid containing content from both the ED and acute care inpatient modules). All modules shared 11 core learning objectives and included an additional 10–16 questions tailored to the specific clinical unit (i.e., ED, acute care inpatient, or pediatric resident hybrid). The core learning objectives covered general sepsis knowledge. Simultaneously, the additional unit-specific questions familiarized the learner with the relevant sepsis tools and processes introduced as part of the QI initiative. We assigned the same module to all learners (bedside nurses, advanced practitioners, and physicians) within the same clinical unit to promote a unified approach amongst care-team members. The modules presented questions in a multiple-choice format using various question stems, including basic tests of knowledge, advanced applications of concepts and best practices, and interactive case descriptions that required learners to identify the best course of action (**see Appendix A, Panels 1, 2, and 3,** Panel 1. Screenshots of one question from the eLearning module in vivo displaying functionality with the incorporation of confidence-weighted testing. Learners continue to re-see questions answered incorrectly and/or without confidence later on as they progress through the module until they answer all questions correctly and confidently. Panel 2. Example of complete educational content for one question (same as Panel 1) from the eLearning module, including question stem and learning objectives for correct and incorrect selections. Panel 3. Sample of five question stems from combined ED and acute care module. Panel 4. *Previously published in Pediatric Quality & Safety as meeting proceeding (doi: 10.1097/pq9.0000000000 000159):* “Heat map showing the distribution of confidently held misinformation (CHM) and learner Struggle. Areas of highest CHM and Struggle are highlighted with a red circle. […] For the CHM heat map on the left, individual questions are on the horizontal axis (from highest to lowest CHM, left to right) and individual learners on the vertical axis (from lowest to highest CHM, bottom to top). […] For Struggle, individual questions are on the horizontal axis (from highest to lowest Struggle, left to right) and individual learners on the vertical axis (from lowest to highest Struggle, bottom to top)”^1^, **Supplemental Digital Content 1,** http://links.lww.com/PQ9/A299).

Each question first diagnosed the learner’s mastery of its learning objective before providing any educational content; asking questions before providing instruction prepares the brain to learn.^13^ By presenting the questions in multiple-choice format, we could provide instruction on incorrect and correct responses, which provided additional educational opportunities beyond the question’s specific learning objective.^14^ The novelty of this eLearning intervention lies in the incorporation of confidence-weighted testing; learners identified their confidence level in each answer choice (“I am sure,” “I am unsure,” or “I don’t know yet”). Confidence-weighted testing improves learning of the material and increases learners’ knowledge of educational content tied to both correct and incorrect answer choices for each question.^10,11^ Additionally, it allows for individualized feedback to optimize the learner’s time and knowledge acquisition. For example, when a learner confidently answers a question correctly, no additional instruction or corrective feedback is provided during the module because it would not improve learning.^15^ Conversely, low-confidence correct answers and incorrect answers are accompanied by delayed instruction and corrective feedback, which improves learning.^16,17^ All questions with incorrect or unsure responses were repeated throughout the module until Amplifire’s algorithms determined that mastery had been achieved. The algorithms managed the delays between attempt and correction and between repetition of the question to allow as much forgetting as possible without making the question impossible to answer again.^18^ This approach optimizes the cognitive benefit from the delay between learning events.^19^ From the nature of the learner’s responses over time, the algorithms determine whether mastery has been achieved; in all cases, each question is answered confidently and correctly at least once.^20^

Previously hired bedside nurses, advanced practitioners (nurse practitioners and physician’s assistants), attending physicians, and pediatric residents in the ED and acute care inpatient units completed the module in January 2018. New residents and newly hired nurses, advanced practitioners, and attending physicians subsequently completed the module on an ongoing basis through December 2019. The module was not distributed to staff in ICUs.

### Data Source and Measures

We collected completion and response data for 2 years from January 1, 2018, through December 31, 2019. We obtained learner demographic and module completion data directly from the institutional LMS, and Amplifire provided response data.

Learner demographic data included role (bedside nurse, advanced practitioner, attending physician, or pediatric resident), work location (free-standing children’s hospital or community hospital), and work unit (ED, acute care inpatient unit, or both). The “both” work unit corresponded to the hybrid module provided to the pediatric residents. We combined advanced practitioners and physicians into a single “provider” role variable for the logistic and linear regression analyses described below.

We treated module completion as a dichotomous (yes/no) variable for each learner.

Response data included Amplifire metrics that consider both the accuracy and level of confidence of learner responses to individual questions (Table 1). We specifically evaluated three: CHM, struggle, and mastery. *CHM* occurs when a learner’s initial response to a question is confident and wrong. The percentage of questions a learner encounters initially in a CHM state defines that learner’s *% CHM*. This metric also describes content; the percentage of *learners* who are initially in a CHM state defines a *question’s* % CHM. Learner struggle occurs when a learner is not initially correct and/or confident, *and* the pedagogical feedback does not cause the learner to become correct and confident on the next attempt. In our analysis, we defined *struggle* as any time a learner has one or more *nonprogress attempts*. As with CHM, we determined *% struggle*, which can describe a question or a learner. *Mastery* occurs when a learner’s initial response to a question is confident and correct. Like CHM and struggle, the *% mastery* can describe a question or a learner. Completion of the module requires ultimately answering all questions correctly and with confidence. We treated all three of these metrics *(% CHM, % struggle,* and *% mastery*) as continuous variables between 0% and 100%.

**Table 1.** Description of Amplifire Metrics

*CHM*: A learner answered confidently and incorrectly the first time presented with the question
*Significant struggle:* Learner answered incorrectly or with uncertainty at least twice before mastering the question (1+ nonprogress attempts)
*Mastery*: A learner answered confidently and correctly the first time presented with the question

Each metric can be summarized at the learner level (eg, the proportion of questions answered by an individual learner that fulfilled metric criteria) and the question level (eg, the proportion of learners for an individual question that fulfilled metric criteria).

### Analysis

We evaluated associations between demographics and module completion using descriptive and inferential statistics, including chi-square tests. We excluded the 140 pediatric residents from the subsequent analyses because they worked throughout the hospital system, and so we could not stratify their data by work unit or location. We performed multivariable logistic regression analyses to evaluate the association of learner work unit, role, and work location with module completion.

The distributions of % CHM, % struggle, and % mastery were visualized as histograms, which indicated nonnormality. The percentage of questions per learner demonstrating CHM, struggle, and mastery were then summarized and stratified by learner work unit, role, and work location as medians with interquartile ranges. The stratified populations were compared using Mann–Whitney U tests. We evaluated the association between learner work unit, role, work location, and % CHM, % struggle, and % mastery using linear regression analysis.

## RESULTS

### Module Completion

In total, 83% of assigned care-team members completed the modules (1,463/1,754) between January 1, 2018, and December 31, 2019. Learners required a mean of 29 minutes to complete the ED module, 25 minutes for the resident hybrid module, and 24 minutes for the acute care inpatient module. Each learner saw each item a mean of 1.72 times before the algorithms determined that mastery had been attained.

Table 2 shows the learner pool and module completion characteristics, stratified by work unit (ED, acute care inpatient, or both), role, and work location (free-standing children’s hospital or community hospital). For ED, 93% of assigned care-team members completed their assigned module (402/433), including 89% of physicians (94/106), 88% of advanced practitioners (56/64), and 96% of nurses (252/263). For acute care inpatient, 82% of assigned care-team members completed their assigned module (974/1,181), including 52% of physicians (141/270), 58% of advanced practitioners (80/138), and 97% of nurses (753/773). For pediatric residents, 62% completed their assigned module (87/140).

**Table 2.** Characteristics of Learner Pool and Module Completion Stratified by Work Unit, Role, and Work Location

Learner Demographics	All Assigned, n (%)	Did Not Complete, n (%)	Completed, n (%)	*P*
Total modules assigned	1,754 (100)	291 (100)	1,463 (100)	N/A
Work unit				<0.001
ED	433 (25)	31 (11)	402 (27)	
Acute care inpatient	1,181 (67)	207 (70)	974 (67)	
Both (pediatric resident hybrid)*	140 (8)	53 (18)	87 (62)	
Role				<0.001
Bedside nurse	1,036 (59)	31 (11)	1,005 (69)	
Advanced practitioner†	202 (12)	66 (23)	136 (9)	
Attending physician†	376 (21)	141 (48)	235 (16)	
Pediatric resident*	140 (8)	53 (18)	87 (6)	
Work location				<0.001
Free-standing children’s hospital	1,406 (80)	267 (92)	1,139 (78)	
Community hospital	348 (20)	24 (8)	324 (93)	

*Excluded from regressions.

†Combined as “provider” in regressions.

Module completion was higher in the ED (93%) than in acute care inpatient units (82%) and for bedside nurses (97%) than for providers (64%). These results remain statistically significant after adjusting for covariates in multivariable logistic regression analyses, including all 1,614 eligible nonresident learners in the ED and acute care inpatient units. Care-team members who completed their assigned module had four times the odds of being from the ED than from acute care inpatient units (aOR 3.8, *P* < 0.001) and 20 times the odds of being a bedside nurse than a provider (aOR 20.0, *P* < 0.001). We found no significant association between work location and completion.

### Learner Responses

A total of 1,376 nonresident learners from ED and non-ICU units completed their assigned modules (Table 2), of whom 1,320 (96%) had a complete response and demographic data after merging our two data sources. Table 3 shows comparisons of median % CHM, % mastery, and % struggle between demographic groups for those learners with a complete response and demographic dataset.

**Table 3.** Comparison of Median % CHM, % Struggle, and % Mastery*

Learner Demographics	n (%)	% CHM, Median (IQR)	*P*	% Struggle, Median (IQR)	*P*	% Mastery, Median (IQR)	*P*
Total	1,320 (100)	9.5 (4.8, 17.4)	—	0 (0, 4.8)	—	61.9 (42.9, 76.1)	—
Work unit			<0.001		0.41		<0.001
ED	388 (29)	13.0 (8.7, 21.7)		4.4 (0, 8.7)		56.5 (39.1, 69.6)	
Acute care inpatient	932 (71)	9.5 (4.8, 14.3)		0 (0, 4.8)		61.9 (42.9, 76.2)	
Role			0.48		<0.001		<0.001
Bedside nurse	946 (72)	9.5 (4.8, 17.4)		4.4 (0, 4.8)		57.1 (39.1, 71.4)	
Provider	374 (28)	9.5 (4.8, 14.3)		0 (0, 4.4)		69.6 (52.4, 81.0)	
Work location			<0.001		0.055		0.21
Free-standing children’s hospital	1010 (77)	9.5 (4.8, 14.3)		0 (0, 4.8)		61.9 (42.9, 76.2)	
Community hospital	310 (23)	14.3 (4.8, 21.7)		4.4 (0, 8.7)		60.1 (42.9, 73.9)	

*Median values were calculated by determining the individual % for each learner (the proportion of questions answered by an individual learner that fulfilled metric criteria) and then calculating the median % across all learners in a given subgroup.

The linear regression analysis revealed that % CHM was higher by 5.8% for ED compared to acute care inpatient unit care-team members (95% CI 4.7–6.9), 1.3% for bedside nurses compared to providers (95% CI 0.2–2.4), and 1.8% for community hospital compared to free-standing children’s hospital care-team members (95% CI 0.6–3.0).

The % struggle was higher by 1.5% for ED than acute care inpatient unit care-team members (95% CI 0.8–2.3) and 2.7% for bedside nurses compared to providers (95% CI 2.0–3.5). We found no significant relationship between % struggle and work location.

The % mastery was higher by 8.3% for acute care inpatient unit compared to ED care-team members (95% CI 5.6–10.9), 12.2% for providers compared to bedside nurses (95% CI 9.6–14.8), and 3.1% for community hospital compared to free-standing children’s hospital care-team members (95% CI 0.25–6.0).

**Fig. 1.**
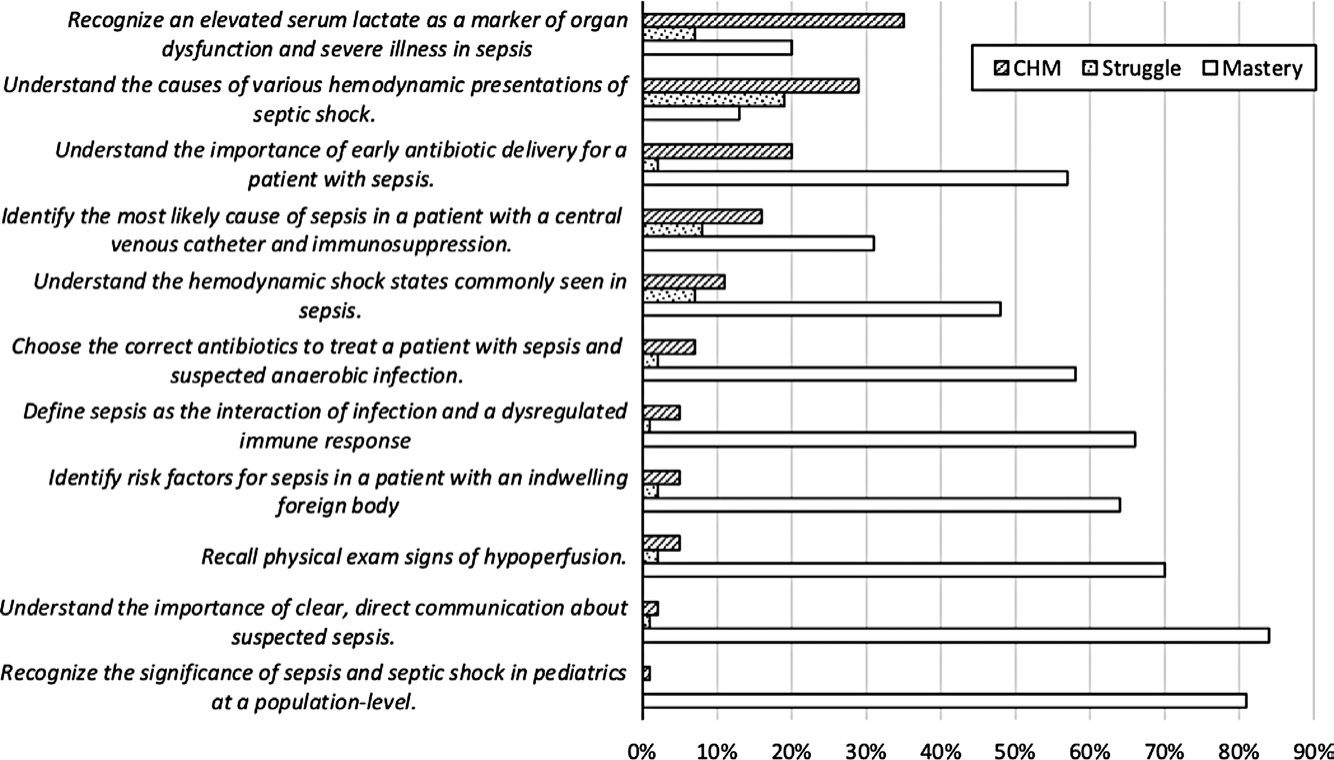
Core learning objectives common to all three sepsis modules with the % of learners fulfilling criteria for CHM, learner struggle, and mastery.

Figure 1 shows the core questions’ learning objectives common to all 3 modules, along with their associated CHM and struggle. As shown in the figure, there was overlap in CHM and struggle, with areas of highest CHM and struggle centered around (1) the misconception that tourniquet use in routine blood draws creates substantial false elevations in lactate and (2) awareness of the hemodynamic shock states commonly seen in sepsis. Additionally, high CHM was seen in understanding the importance of early antibiotic delivery in patients with septic shock.

## DISCUSSION

This project successfully provided education to frontline care-team members as part of a QI initiative using educational theory to optimize its impact. We simultaneously identified opportunities for ongoing improvement and focused education using eLearning enhanced by confidence-weighted testing. This approach optimized knowledge attainment and skill acquisition for the individual learner by providing real-time feedback based on the learner’s knowledge and confidence, which translates to the correction of the learner’s deficits and improved knowledge retention.

Although this study is cross-sectional rather than longitudinal, the eLearning platform collects data over time in the learner’s path to knowledge acquisition for each learning objective. The % mastery across all questions for all learners’ initial attempts was 62%, suggesting that the learners began the module with an incomplete sepsis knowledge base. Upon completion of the module, all learners had answered 100% of the questions correctly and with full confidence at least once. This result indicates significant knowledge acquisition resulting from the completion of our module.

Another strength of this intervention is its relatively short time to complete (less than 30 minutes on average for all module variants). This finding ensures the modules’ accessibility to busy bedside nurses, advanced practitioners, attending physicians, and pediatric residents.

Differing strategies used to distribute the module assignments may explain the higher completion rates in bedside nurses than other care-team members seen in Table 2. Although we assigned the modules to the entire learner pool using the same LMS, messaging to nurses labeled it mandatory while other provider groups resisted that word. There may also be inherent differences between the groups, perhaps in their perceptions of the usefulness of institutional education, responses to authority, and/or eagerness to learn. Providers may also have felt they had less to gain from completing the modules; the higher mastery and lower struggle seen for providers than bedside nurses support that hypothesis.

We observed higher CHM and struggle in bedside nurses than providers, highlighting a potential need to educate bedside nurses further. This observation may be driven by differences between groups in their experiences and training before the intervention. We also observed higher CHM and struggle in ED compared to acute care inpatient care-team members. This observation is a surprising outcome given the greater frequency at which ED care-team members see patients with sepsis and suspected sepsis compared to acute care inpatient providers. A possible explanation for this finding is that, because they have more real-life experience with sepsis in their clinical practice, ED care-team members may feel more confident in their competence in this area—even when that confidence is misplaced.

Figure 1 shows an advantage of employing confidence-weighted testing to identify firmly held but misdirected beliefs that may be potential sources of near misses and adverse events. The third row of data shows that 57% of learners demonstrated mastery on their first attempt at questions about the importance of early antibiotic delivery for patients with sepsis. The fourth row shows that far fewer learners (31%) had initial mastery regarding causes of sepsis in patients with central venous catheters. Suppose initial mastery was the only determinant of the need for intervention. In that case, one might believe that behavior involving central venous catheters was of greater concern than early antibiotic delivery. But the CHM data reveal otherwise, as fewer learners had CHM about central venous catheters (16%) than antibiotics (20%). Although learners started the module with a more significant knowledge gap around central venous catheters, that was easier to correct than the antibiotics’ misinformation. In this way, by identifying areas of highest CHM, our project can help inform future interventions within our broader institutional sepsis initiative (i.e., learning points found in the areas of highest CHM can be re-addressed in other forms in subsequent interventions).

Other studies have similarly shown that eLearning can improve sepsis knowledge.^7^ One sepsis team demonstrated knowledge gain from their eLearning intervention and improvements in attitude and behavior regarding sepsis among their pediatric providers.^6^ Our study adds to these findings by showing the additional benefits of confidence-weighted testing in knowledge gain, skill acquisition, and prioritization of institutional improvement efforts. eLearning allows for individualization of the path to knowledge acquisition within each learner’s experience, identifying individual learners in need of focused intervention/remediation, and identifying institutional problem areas as opportunities for targeted QI interventions. Confidence-weighted testing further enhances eLearning by identifying where misconceptions are more likely to become mistakes.

A limitation of this project is the wide range in module completion rates among the targeted audience. Further effort is needed to improve completion rates, especially among providers. The lack of pretest and posttest makes it challenging to compare this to other studies or demonstrate the modules’ impact on learner performance and knowledge acquisition. Our study’s cross-sectional design and the simultaneous implementation of competing interventions as part of the broader sepsis improvement initiative prohibit us from isolating this educational module’s effect on patient outcomes over time. Additionally, we did not correlate questions within a single learner, so specific learners may drive the associations observed for a single group based on that learner’s tendency to answer all questions in a certain way.

## CONCLUDING SUMMARY

This QI intervention uses a novel approach to eLearning. We showed that education could effectively be provided to a diverse population (including nurses, physicians, and advanced practitioners) using a unified approach. The use of confidence-weighted testing allowed us to identify differences in knowledge base and acquisition between different hospital units and learner roles. We simultaneously provided education on pediatric sepsis topics while also identifying areas where learners had difficulty changing their beliefs when misguided. This CHM represents areas of risk within the system and opportunities for subsequent improvement efforts.

## DISCLOSURE

Dr Hays is an employee of Amplifire, Inc., the proprietary eLearning company with which we collaborated to develop the intervention described herein. Amplifire, Inc. provides Children’s Hospital Colorado with a portion of revenue from any licenses sold for modules it helps create, but none of the authors benefit from this arrangement. The remaining authors do not have any individual conflicts of interest or financial disclosures. The other authors have no financial interest to declare in relation to the content of this article.

## Supplementary Material


