# Supplementary material for: Confidence-weighted Testing as an Impactful Education Intervention within a Pediatric Sepsis Quality Improvement Initiative
Source: Pediatr Qual Saf. 2021 Aug 26;6(5):e460. doi: 10.1097/pq9.0000000000000460 (PMC8389944; doi:10.1097/pq9.0000000000000460)
Supplement: Supplementary file 1 [file pqs-6-e460-s001.pdf]

Appendix A.

Panel 1. Screenshots of one question from the eLearning module *in vivo* displaying functionality with the incorporation of confidence-weighted testing. Learners continue to re-see questions answered incorrectly and/or without confidence later on as they progress through the module until they answer all questions correctly and confidently.

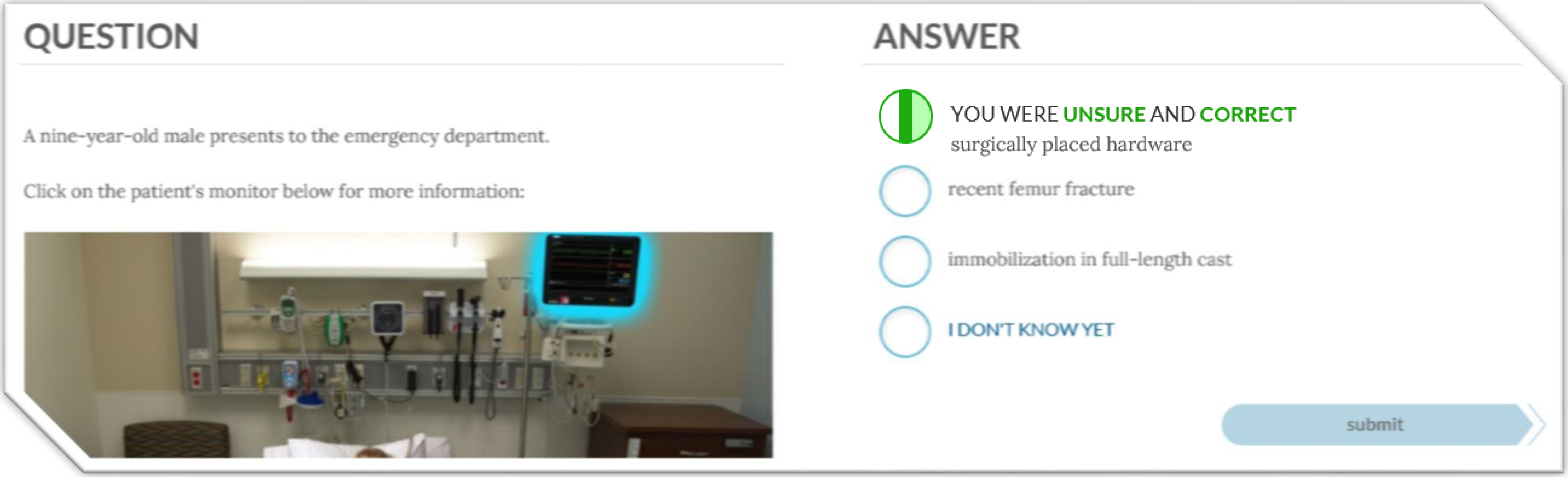

Panel 2. Example of complete educational content for one question (same as Panel 1) from the eLearning module, including question stem and learning objectives for correct and incorrect selections.

<u>Question</u>

A nine-year-old male presents to the emergency department. Click on the patient's monitor below for more information:

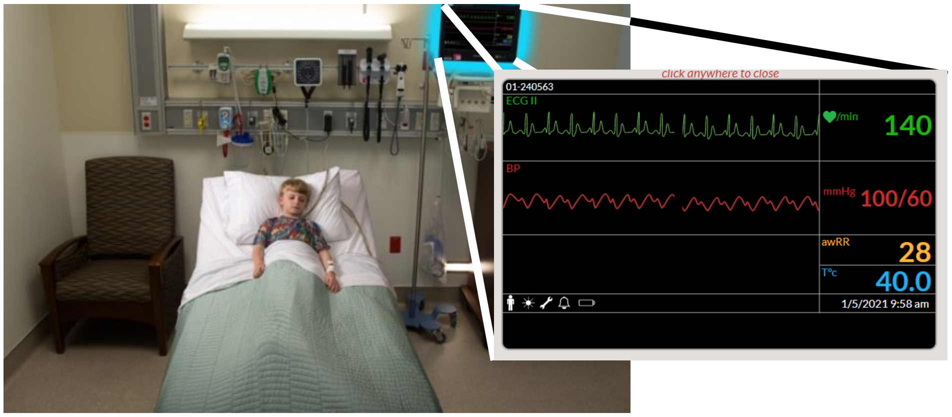

On exam he is tearful and clinging to his mother. He recently underwent pinning of a closed fracture of the left femur and is in a full-length lower-extremity cast. His mother reports that he has been complaining of increasing pain at his surgery site. *You suspect sepsis because of __________.*

<u>Answer choices</u>

A. Recent femur fracture
B. Surgically placed hardware (correct)
C. Immobilization in full-length case

<u>"What you need to know"</u>

Surgically placed hardware, intra-abdominal abscesses, necrotizing fasciitis, and catheters can all become localized sources of infection requiring removal, drainage, or debridement. This patient's indwelling foreign body (pins), combined with his abnormal vital signs (tachycardia and fever), are concerning for sepsis. Surgically placed hardware can serve as a reservoir of infection either immediately after placement or any time later. An infected foreign body can continue to seed infection despite administration of effective antibiotics.

While immobilization may put this patient at risk for thrombus formation, broken bones and casting do not greatly increase the risk for developing sepsis. Open fractures and patients with underlying immunosuppression may have a higher risk of infection at the fracture site.

Panel 3. Sample of five question stems from combined ED & acute care module.

Question	Answer choices
One characteristic of both warm and cold septic shock is relative hypovolemia. What are other characteristics of COLD shock?	A. Vasoconstriction and decreased cardiac output<br>B. Vasodilation and decreased systemic vascular resistance<br>C. Vasoconstriction and high cardiac output
Early recognition is required to deliver life-saving sepsis treatment. Which pediatric sepsis treatment has been shown to save lives if given in a timely manner?	A. Antibiotics<br>B. Intravenous fluid bolus<br>C. Intubation
You suspect sepsis on a febrile patient with a central line. The port would not draw, so a PIV was placed and labs were drawn with a tourniquet. The labs return with a normal white count and a serum lactate of 4. What is significant about the serum lactate result in this patient?	A. Lactate ≥2 mmol/L is associated with increased risk of death and organ dysfunction<br>B. Serum lactate will always be elevated in critically ill septic patients<br>C. Lactate levels will be falsely elevated in critically ill septic patients
Gram-positive infections are more typically associated with __________.	A. Cold shock<br>B. Warm shock<br>C. Immunocompromised<br>D. Brisk capillary refill
Acute Care: A four-year-old male was admitted 14 hours ago with coughing and vomiting for the past five days thought to be secondary to community-acquired pneumonia. He has no history of asthma and reports pain when taking deep breaths. He is fully vaccinated and has no prior medical history. He has had rising tachycardia and intermittent fevers since admission. Exam: Patient is tired, arouses for exam but falls asleep when not answering questions. He has notable abdominal breathing and retractions. Pulses are weak, and extremities are mottled. Capillary refill is four seconds. *Click on the patient's monitor below for more information (HR 180, BP 68/30, T 40°C).* An RRT is called due to suspected sepsis with hypotension and impaired perfusion. While awaiting the arrival of the RRT, the Inpatient Suspected Sepsis order set is opened. Which of the following are priorities in a patient with suspected sepsis?	A. IV access, IV antibiotics, volume resuscitation with signs of hypovolemia, and continuous monitoring<br>B. Antipyretics, cooling blanket, and routine vital signs<br>C. Continuous albuterol, systemic steroids, and aggressive suctioning

Panel 4. *Previously published in Pediatric Quality & Safety as meeting proceeding (doi: 10.1097/pq9.0000000000000159): "*Heat map showing the distribution of confidently held misinformation (CHM) and learner Struggle. Areas of highest CHM and Struggle are highlighted with a red circle. […] For the CHM heat map on the left, individual questions are on the horizontal axis (from highest to lowest CHM, left to right) and individual learners on the vertical axis (from lowest to highest CHM, bottom to top). […] For Struggle, individual questions are on the horizontal axis (from highest to lowest Struggle, left to right) and individual learners on the vertical axis (from lowest to highest Struggle, bottom to top)" [1].

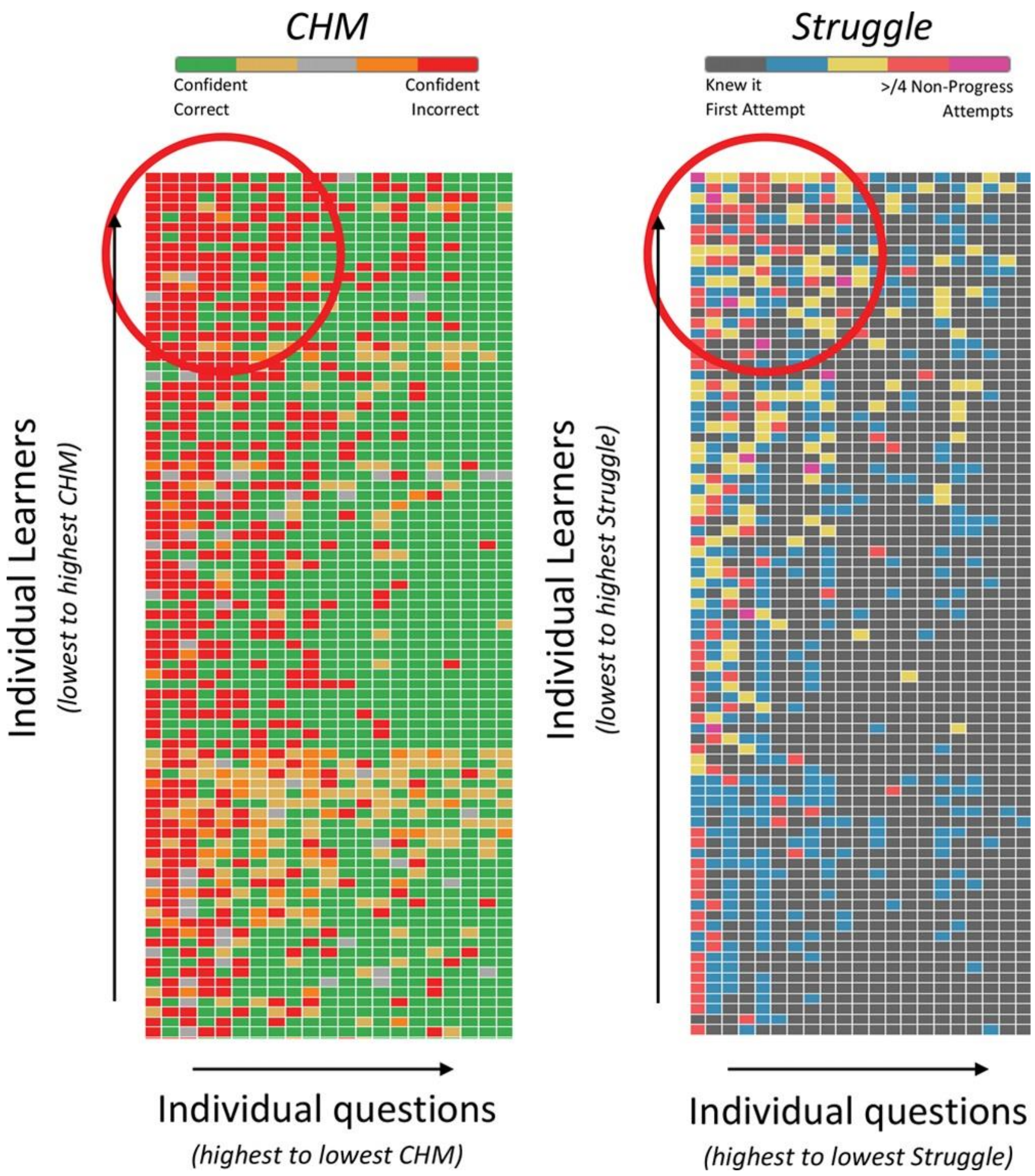

References.

1. Lockwood, J., et al., *"Is This Sepsis?" Education: Leveraging "Confidently Held Misinformation" and Learner "Struggle".* Pediatric Quality & Safety, 2019. **4**(Suppl 3): p. e169.
